# Supplementary material for: Association between Weather Types based on the Spatial Synoptic Classification and All-Cause Mortality in Sweden, 1991–2014
Source: Int J Environ Res Public Health. 2019 May 14;16(10):1696. doi: 10.3390/ijerph16101696 (PMC6573000; doi:10.3390/ijerph16101696)
Supplement: Supplementary file 1 [file ijerph-16-01696-s001.pdf]

*Article - Supplementary Files*

# Association between weather types based on the Spatial Synoptic Classification and all-cause mortality in Sweden, 1991 – 2014

**Osvaldo Fonseca-Rodríguez[1, 2, *], Erling Häggström Lundevaller[2], Scott C. Sheridan[3] and Barbara Schumann[1,2]**

1 Department of Epidemiology and Global Health, Umeå University, Umeå, Sweden; osvaldo.fonseca@umu.se, barbara.schumann@umu.se
2 Centre for Demographic and Ageing Research, Umeå University, Umeå, Sweden; erling.lundevaller@umu.se
3 Department of Geography, Kent State University, Kent, Ohio, USA; ssherid1@kent.edu
* Correspondence: osvaldo.fonseca@umu.se; Tel.: +46-90-786-9813

## Table of Contents

**Figure S1.** Time series of daily number of deaths in each location: Skåne, Stockholm, Jämtland, Västerbotten. Excess deaths in Stockholm related to major disasters were omitted in the graph (237 deaths on 28 September 28 1994 and 275 deaths on 26 December 2004).

**Figure S2.** Sum of monthly number of deaths in each location during the study period. The summer and winter months are highlighted in red and blue, respectively.

**Table S1.** Total quasi Akaike information criterion (qAIC) summed across all sites in **summer** by different combinations of knots and degrees of freedom for the spline function of years and day of the year (DOY). The lowest qAIC is in bold letters and it was for the combination: knots= 3, ns (year, df= 3), ns (DOY, df=3).

**Table S2.** Total quasi Akaike information criterion (qAIC) summed across all sites in **winter** by different combinations of knots and degrees of freedom for the spline function of years and day of the year (DOY). The lowest qAIC is in bold letters and it was for the combination: knots= 3, ns (year, df= 3), ns (DOY, df=3).

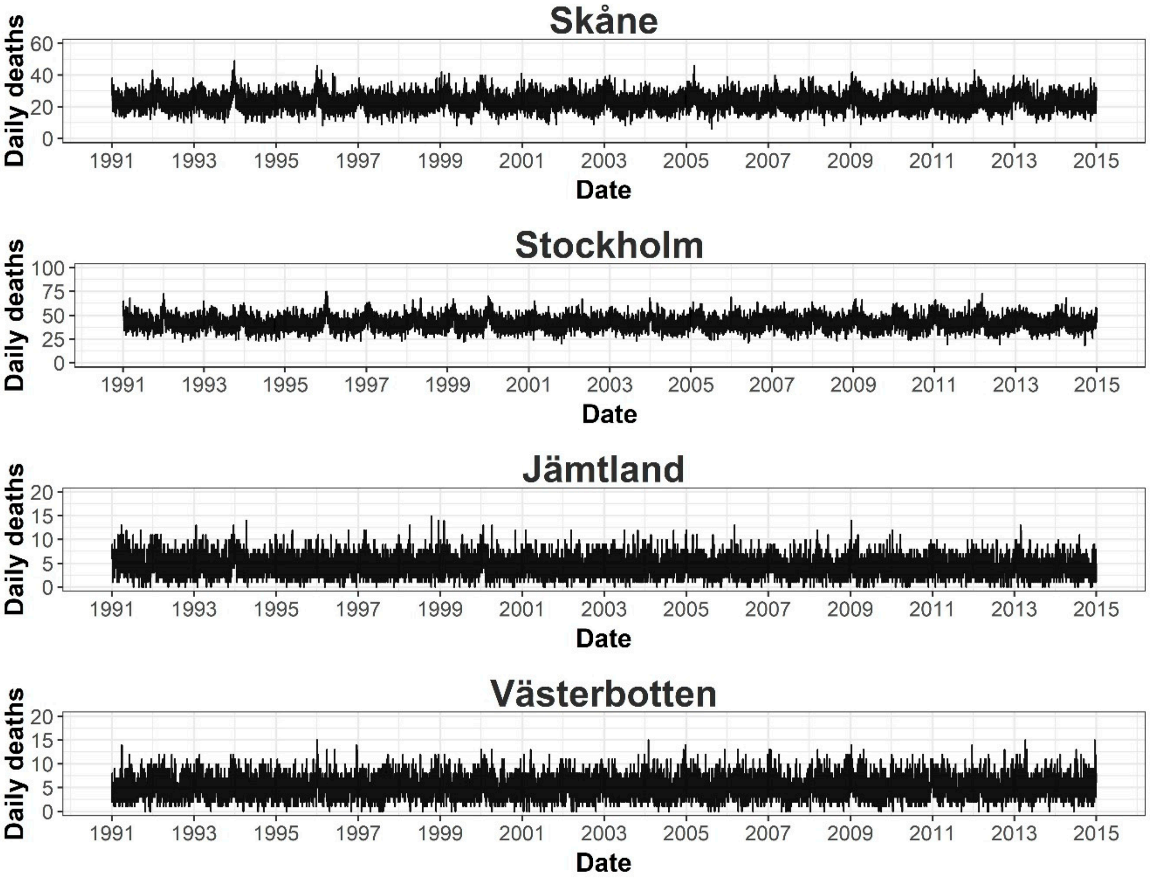


**Figure S1.** Time series of daily number of deaths in each location: Skåne, Stockholm, Jämtland, Västerbotten. Excess deaths in Stockholm related to major disasters were omitted in the graph (237 deaths on 28 September 1994 and 275 deaths on 26 December 2004).

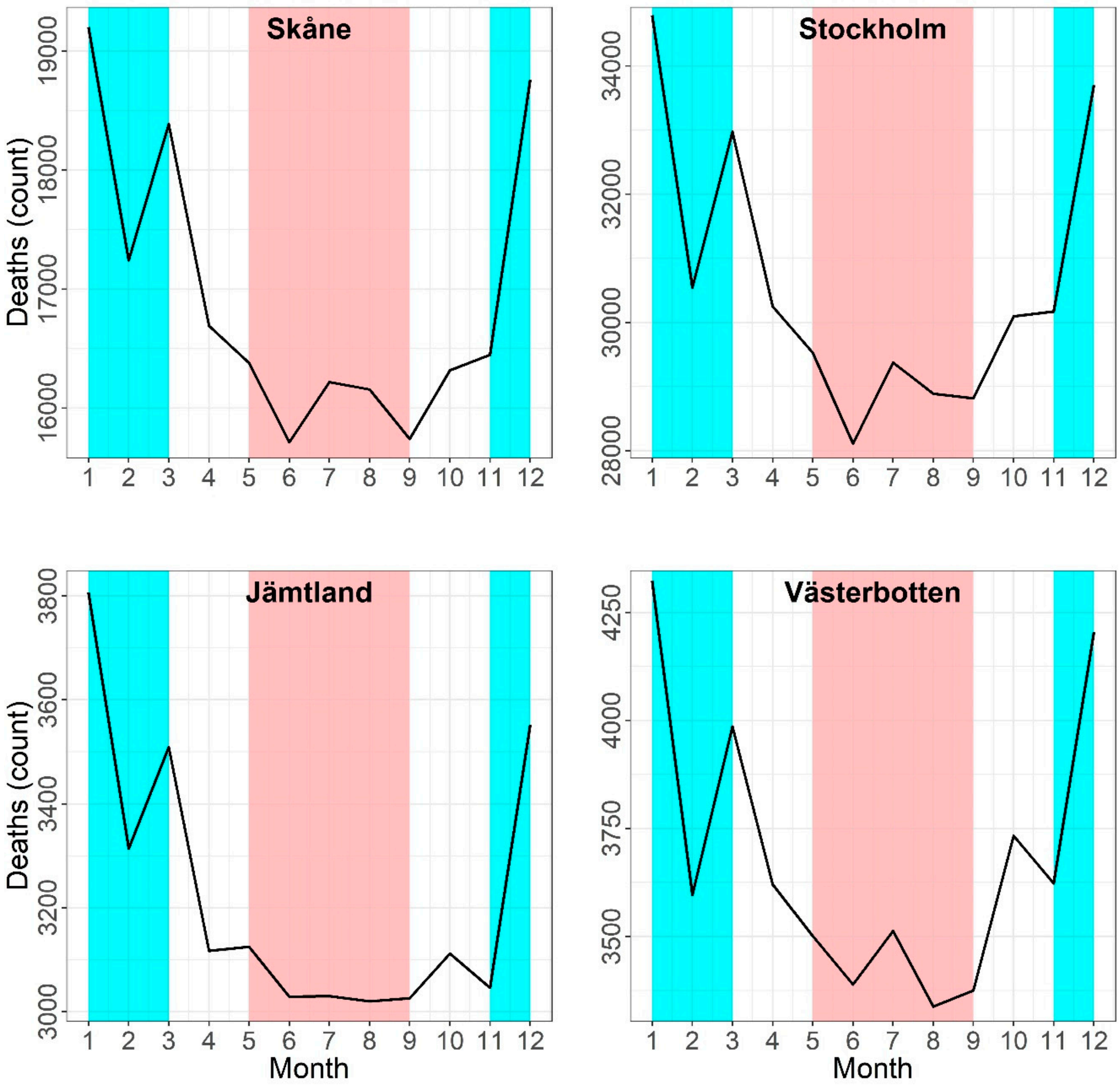


**Figure S2.** Sum of monthly number of deaths in each location during the study period. The summer and winter months are highlighted in red and blue, respectively..

**Table S1.** Total quasi Akaike information criterion (qAIC) summed across all sites in **summer** by different combinations of knots and degrees of freedom for the spline function of years and day of the year (DOY). The lowest qAIC is in bold letters and it was for the combination: knots= 3, ns (year, df= 3), ns (DOY, df=3).

| **Stockholm-Summer** | | | | **Skåne-Summer** | | | | **Jämtland-Summer** | | | | **Västerbotten-Summer** | | | | **Total qAIC summed across all sites** |
|---|---|---|---|---|---|---|---|---|---|---|---|---|---|---|---|---|
| knots | ns (year, df) | ns (DOY, df) | qAIC | knots | ns (year, df) | ns (DOY, df) | qAIC | knots | ns (year, df) | ns (DOY, df) | qAIC | knots | ns (year, df) | ns (DOY, df) | qAIC | |
| 3 | 3 | 3 | 18073.7 | 3 | 3 | 3 | 17523.3 | 3 | 3 | 3 | 12990.5 | 3 | 3 | 3 | 11708.5 | **60296.0** |
| 3 | 3 | 4 | 18084.6 | 3 | 3 | 4 | 17519.9 | 3 | 3 | 4 | 12994.1 | 3 | 3 | 4 | 11703.0 | 60301.7 |
| 3 | 3 | 5 | 18082.4 | 3 | 3 | 5 | 17519.5 | 3 | 3 | 5 | 12992.1 | 3 | 3 | 5 | 11707.0 | 60301.0 |
| 3 | 3 | 6 | 18080.0 | 3 | 3 | 6 | 17526.8 | 3 | 3 | 6 | 12992.7 | 3 | 3 | 6 | 11712.6 | 60312.1 |
| 3 | 3 | 7 | 18079.5 | 3 | 3 | 7 | 17516.4 | 3 | 3 | 7 | 13007.9 | 3 | 3 | 7 | 11722.7 | 60326.6 |
| 3 | 3 | 8 | 18098.0 | 3 | 3 | 8 | 17522.9 | 3 | 3 | 8 | 13004.1 | 3 | 3 | 8 | 11739.0 | 60364.0 |
| 3 | 4 | 3 | 18094.0 | 3 | 4 | 3 | 17530.9 | 3 | 4 | 3 | 13006.2 | 3 | 4 | 3 | 11706.5 | 60337.5 |
| 3 | 4 | 4 | 18103.7 | 3 | 4 | 4 | 17527.4 | 3 | 4 | 4 | 13008.9 | 3 | 4 | 4 | 11701.2 | 60341.2 |
| 3 | 4 | 5 | 18101.4 | 3 | 4 | 5 | 17527.0 | 3 | 4 | 5 | 13006.9 | 3 | 4 | 5 | 11705.0 | 60340.3 |
| 3 | 4 | 6 | 18099.2 | 3 | 4 | 6 | 17534.4 | 3 | 4 | 6 | 13007.3 | 3 | 4 | 6 | 11710.6 | 60351.6 |
| 3 | 4 | 7 | 18098.4 | 3 | 4 | 7 | 17524.0 | 3 | 4 | 7 | 13022.2 | 3 | 4 | 7 | 11720.7 | 60365.3 |
| 3 | 4 | 8 | 18116.8 | 3 | 4 | 8 | 17530.5 | 3 | 4 | 8 | 13018.1 | 3 | 4 | 8 | 11736.8 | 60402.3 |
| 3 | 5 | 3 | 18110.1 | 3 | 5 | 3 | 17540.3 | 3 | 5 | 3 | 13007.9 | 3 | 5 | 3 | 11708.8 | 60367.1 |
| 3 | 5 | 4 | 18118.9 | 3 | 5 | 4 | 17536.7 | 3 | 5 | 4 | 13010.6 | 3 | 5 | 4 | 11703.4 | 60369.7 |
| 3 | 5 | 5 | 18116.6 | 3 | 5 | 5 | 17536.5 | 3 | 5 | 5 | 13008.6 | 3 | 5 | 5 | 11707.7 | 60369.3 |
| 3 | 5 | 6 | 18114.6 | 3 | 5 | 6 | 17543.8 | 3 | 5 | 6 | 13008.9 | 3 | 5 | 6 | 11712.9 | 60380.1 |
| 3 | 5 | 7 | 18113.7 | 3 | 5 | 7 | 17533.3 | 3 | 5 | 7 | 13023.9 | 3 | 5 | 7 | 11723.3 | 60394.1 |
| 3 | 5 | 8 | 18132.0 | 3 | 5 | 8 | 17540.0 | 3 | 5 | 8 | 13019.8 | 3 | 5 | 8 | 11739.2 | 60431.0 |
| 3 | 6 | 3 | 18143.5 | 3 | 6 | 3 | 17553.9 | 3 | 6 | 3 | 13007.1 | 3 | 6 | 3 | 11713.9 | 60418.4 |
| 3 | 6 | 4 | 18152.2 | 3 | 6 | 4 | 17550.4 | 3 | 6 | 4 | 13009.8 | 3 | 6 | 4 | 11708.4 | 60420.8 |
| 3 | 6 | 5 | 18149.8 | 3 | 6 | 5 | 17550.3 | 3 | 6 | 5 | 13007.7 | 3 | 6 | 5 | 11713.0 | 60420.8 |
| 3 | 6 | 6 | 18147.7 | 3 | 6 | 6 | 17557.2 | 3 | 6 | 6 | 13008.0 | 3 | 6 | 6 | 11718.0 | 60431.0 |
| 3 | 6 | 7 | 18147.1 | 3 | 6 | 7 | 17546.7 | 3 | 6 | 7 | 13023.3 | 3 | 6 | 7 | 11728.5 | 60445.6 |
| 3 | 6 | 8 | 18165.5 | 3 | 6 | 8 | 17553.5 | 3 | 6 | 8 | 13019.2 | 3 | 6 | 8 | 11745.0 | 60483.1 |

| | | | | | | | | | | | | | | | | |
|---|---|---|---|---|---|---|---|---|---|---|---|---|---|---|---|---|
| 3 | 7 | 3 | 18149.6 | 3 | 7 | 3 | 17547.6 | 3 | 7 | 3 | 13027.2 | 3 | 7 | 3 | 11719.3 | 60443.7 |
| 3 | 7 | 4 | 18158.6 | 3 | 7 | 4 | 17544.0 | 3 | 7 | 4 | 13029.6 | 3 | 7 | 4 | 11713.9 | 60446.1 |
| 3 | 7 | 5 | 18156.2 | 3 | 7 | 5 | 17543.8 | 3 | 7 | 5 | 13027.5 | 3 | 7 | 5 | 11718.3 | 60445.8 |
| 3 | 7 | 6 | 18154.1 | 3 | 7 | 6 | 17550.8 | 3 | 7 | 6 | 13027.6 | 3 | 7 | 6 | 11723.3 | 60455.8 |
| 3 | 7 | 7 | 18153.5 | 3 | 7 | 7 | 17540.4 | 3 | 7 | 7 | 13042.9 | 3 | 7 | 7 | 11733.7 | 60470.4 |
| 3 | 7 | 8 | 18171.9 | 3 | 7 | 8 | 17547.0 | 3 | 7 | 8 | 13038.4 | 3 | 7 | 8 | 11750.5 | 60507.8 |
| 3 | 8 | 3 | 18146.6 | 3 | 8 | 3 | 17564.8 | 3 | 8 | 3 | 13031.7 | 3 | 8 | 3 | 11725.2 | 60468.3 |
| 3 | 8 | 4 | 18155.7 | 3 | 8 | 4 | 17561.3 | 3 | 8 | 4 | 13034.1 | 3 | 8 | 4 | 11719.9 | 60471.0 |
| 3 | 8 | 5 | 18153.4 | 3 | 8 | 5 | 17561.5 | 3 | 8 | 5 | 13031.9 | 3 | 8 | 5 | 11724.1 | 60470.9 |
| 3 | 8 | 6 | 18151.4 | 3 | 8 | 6 | 17568.2 | 3 | 8 | 6 | 13031.6 | 3 | 8 | 6 | 11729.2 | 60480.5 |
| 3 | 8 | 7 | 18150.6 | 3 | 8 | 7 | 17557.7 | 3 | 8 | 7 | 13046.5 | 3 | 8 | 7 | 11739.2 | 60494.1 |
| 3 | 8 | 8 | 18169.2 | 3 | 8 | 8 | 17564.6 | 3 | 8 | 8 | 13041.9 | 3 | 8 | 8 | 11756.1 | 60531.7 |
| 4 | 3 | 3 | 18087.2 | 4 | 3 | 3 | 17582.7 | 4 | 3 | 3 | 12991.5 | 4 | 3 | 3 | 11720.1 | 60381.4 |
| 4 | 3 | 4 | 18096.9 | 4 | 3 | 4 | 17578.7 | 4 | 3 | 4 | 12995.8 | 4 | 3 | 4 | 11714.5 | 60385.9 |
| 4 | 3 | 5 | 18094.6 | 4 | 3 | 5 | 17578.2 | 4 | 3 | 5 | 12994.0 | 4 | 3 | 5 | 11718.7 | 60385.5 |
| 4 | 3 | 6 | 18092.3 | 4 | 3 | 6 | 17583.8 | 4 | 3 | 6 | 12994.2 | 4 | 3 | 6 | 11724.5 | 60394.8 |
| 4 | 3 | 7 | 18092.2 | 4 | 3 | 7 | 17573.8 | 4 | 3 | 7 | 13008.7 | 4 | 3 | 7 | 11735.6 | 60410.3 |
| 4 | 3 | 8 | 18110.5 | 4 | 3 | 8 | 17581.0 | 4 | 3 | 8 | 13004.9 | 4 | 3 | 8 | 11752.5 | 60449.0 |
| 4 | 4 | 3 | 18106.7 | 4 | 4 | 3 | 17590.0 | 4 | 4 | 3 | 13007.0 | 4 | 4 | 3 | 11718.3 | 60421.9 |
| 4 | 4 | 4 | 18115.2 | 4 | 4 | 4 | 17585.9 | 4 | 4 | 4 | 13010.4 | 4 | 4 | 4 | 11712.8 | 60424.4 |
| 4 | 4 | 5 | 18112.8 | 4 | 4 | 5 | 17585.4 | 4 | 4 | 5 | 13008.6 | 4 | 4 | 5 | 11716.9 | 60423.8 |
| 4 | 4 | 6 | 18110.8 | 4 | 4 | 6 | 17591.2 | 4 | 4 | 6 | 13008.7 | 4 | 4 | 6 | 11722.6 | 60433.3 |
| 4 | 4 | 7 | 18110.3 | 4 | 4 | 7 | 17581.1 | 4 | 4 | 7 | 13022.8 | 4 | 4 | 7 | 11733.8 | 60448.0 |
| 4 | 4 | 8 | 18128.7 | 4 | 4 | 8 | 17588.5 | 4 | 4 | 8 | 13018.7 | 4 | 4 | 8 | 11750.4 | 60486.3 |
| 4 | 5 | 3 | 18122.2 | 4 | 5 | 3 | 17599.0 | 4 | 5 | 3 | 13008.6 | 4 | 5 | 3 | 11720.8 | 60450.6 |
| 4 | 5 | 4 | 18129.9 | 4 | 5 | 4 | 17594.8 | 4 | 5 | 4 | 13012.0 | 4 | 5 | 4 | 11715.3 | 60452.0 |
| 4 | 5 | 5 | 18127.5 | 4 | 5 | 5 | 17594.5 | 4 | 5 | 5 | 13010.0 | 4 | 5 | 5 | 11719.9 | 60452.0 |
| 4 | 5 | 6 | 18125.6 | 4 | 5 | 6 | 17600.1 | 4 | 5 | 6 | 13010.1 | 4 | 5 | 6 | 11725.2 | 60461.0 |
| 4 | 5 | 7 | 18125.1 | 4 | 5 | 7 | 17590.0 | 4 | 5 | 7 | 13024.4 | 4 | 5 | 7 | 11736.6 | 60476.1 |

| | | | | | | | | | | | | | | | | |
|---|---|---|---|---|---|---|---|---|---|---|---|---|---|---|---|---|
| 4 | 5 | 8 | 18143.3 | 4 | 5 | 8 | 17597.5 | 4 | 5 | 8 | 13020.2 | 4 | 5 | 8 | 11753.2 | 60514.2 |
| 4 | 6 | 3 | 18155.5 | 4 | 6 | 3 | 17612.5 | 4 | 6 | 3 | 13007.9 | 4 | 6 | 3 | 11725.4 | 60501.3 |
| 4 | 6 | 4 | 18163.0 | 4 | 6 | 4 | 17608.4 | 4 | 6 | 4 | 13011.4 | 4 | 6 | 4 | 11719.8 | 60502.6 |
| 4 | 6 | 5 | 18160.5 | 4 | 6 | 5 | 17608.3 | 4 | 6 | 5 | 13009.5 | 4 | 6 | 5 | 11724.7 | 60502.9 |
| 4 | 6 | 6 | 18158.6 | 4 | 6 | 6 | 17613.5 | 4 | 6 | 6 | 13009.5 | 4 | 6 | 6 | 11729.9 | 60511.4 |
| 4 | 6 | 7 | 18158.3 | 4 | 6 | 7 | 17603.4 | 4 | 6 | 7 | 13024.0 | 4 | 6 | 7 | 11741.3 | 60527.0 |
| 4 | 6 | 8 | 18176.6 | 4 | 6 | 8 | 17611.0 | 4 | 6 | 8 | 13019.8 | 4 | 6 | 8 | 11758.4 | 60565.8 |
| 4 | 7 | 3 | 18161.4 | 4 | 7 | 3 | 17605.9 | 4 | 7 | 3 | 13027.9 | 4 | 7 | 3 | 11730.7 | 60526.0 |
| 4 | 7 | 4 | 18169.3 | 4 | 7 | 4 | 17601.8 | 4 | 7 | 4 | 13031.0 | 4 | 7 | 4 | 11725.1 | 60527.2 |
| 4 | 7 | 5 | 18166.8 | 4 | 7 | 5 | 17601.5 | 4 | 7 | 5 | 13029.0 | 4 | 7 | 5 | 11730.0 | 60527.2 |
| 4 | 7 | 6 | 18164.8 | 4 | 7 | 6 | 17606.8 | 4 | 7 | 6 | 13028.8 | 4 | 7 | 6 | 11735.1 | 60535.5 |
| 4 | 7 | 7 | 18164.6 | 4 | 7 | 7 | 17596.8 | 4 | 7 | 7 | 13043.4 | 4 | 7 | 7 | 11746.4 | 60551.1 |
| 4 | 7 | 8 | 18182.9 | 4 | 7 | 8 | 17604.2 | 4 | 7 | 8 | 13038.9 | 4 | 7 | 8 | 11763.8 | 60589.8 |
| 4 | 8 | 3 | 18158.2 | 4 | 8 | 3 | 17623.3 | 4 | 8 | 3 | 13032.0 | 4 | 8 | 3 | 11736.9 | 60550.5 |
| 4 | 8 | 4 | 18166.2 | 4 | 8 | 4 | 17619.2 | 4 | 8 | 4 | 13035.1 | 4 | 8 | 4 | 11731.5 | 60552.1 |
| 4 | 8 | 5 | 18163.9 | 4 | 8 | 5 | 17619.4 | 4 | 8 | 5 | 13033.0 | 4 | 8 | 5 | 11736.0 | 60552.3 |
| 4 | 8 | 6 | 18162.0 | 4 | 8 | 6 | 17624.4 | 4 | 8 | 6 | 13032.5 | 4 | 8 | 6 | 11741.3 | 60560.2 |
| 4 | 8 | 7 | 18161.5 | 4 | 8 | 7 | 17614.4 | 4 | 8 | 7 | 13046.7 | 4 | 8 | 7 | 11752.3 | 60574.8 |
| 4 | 8 | 8 | 18180.0 | 4 | 8 | 8 | 17622.0 | 4 | 8 | 8 | 13042.0 | 4 | 8 | 8 | 11769.7 | 60613.7 |
| 5 | 3 | 3 | 18080.2 | 5 | 3 | 3 | 17615.2 | 5 | 3 | 3 | 12993.7 | 5 | 3 | 3 | 11720.5 | 60409.6 |
| 5 | 3 | 4 | 18089.7 | 5 | 3 | 4 | 17611.3 | 5 | 3 | 4 | 12998.0 | 5 | 3 | 4 | 11715.0 | 60413.9 |
| 5 | 3 | 5 | 18087.4 | 5 | 3 | 5 | 17611.0 | 5 | 3 | 5 | 12996.3 | 5 | 3 | 5 | 11719.1 | 60413.7 |
| 5 | 3 | 6 | 18085.3 | 5 | 3 | 6 | 17616.8 | 5 | 3 | 6 | 12996.4 | 5 | 3 | 6 | 11725.1 | 60423.7 |
| 5 | 3 | 7 | 18085.6 | 5 | 3 | 7 | 17606.7 | 5 | 3 | 7 | 13010.8 | 5 | 3 | 7 | 11736.5 | 60439.6 |
| 5 | 3 | 8 | 18103.7 | 5 | 3 | 8 | 17613.5 | 5 | 3 | 8 | 13007.4 | 5 | 3 | 8 | 11753.2 | 60477.8 |
| 5 | 4 | 3 | 18100.0 | 5 | 4 | 3 | 17622.7 | 5 | 4 | 3 | 13009.6 | 5 | 4 | 3 | 11718.9 | 60451.3 |
| 5 | 4 | 4 | 18108.3 | 5 | 4 | 4 | 17618.7 | 5 | 4 | 4 | 13012.9 | 5 | 4 | 4 | 11713.6 | 60453.5 |
| 5 | 4 | 5 | 18105.9 | 5 | 4 | 5 | 17618.5 | 5 | 4 | 5 | 13011.2 | 5 | 4 | 5 | 11717.6 | 60453.1 |
| 5 | 4 | 6 | 18104.1 | 5 | 4 | 6 | 17624.4 | 5 | 4 | 6 | 13011.2 | 5 | 4 | 6 | 11723.5 | 60463.2 |

| | | | | | | | | | | | | | | | | |
|---|---|---|---|---|---|---|---|---|---|---|---|---|---|---|---|---|
| 5 | 4 | 7 | 18104.0 | 5 | 4 | 7 | 17614.2 | 5 | 4 | 7 | 13025.2 | 5 | 4 | 7 | 11734.9 | 60478.4 |
| 5 | 4 | 8 | 18122.1 | 5 | 4 | 8 | 17621.2 | 5 | 4 | 8 | 13021.5 | 5 | 4 | 8 | 11751.4 | 60516.3 |
| 5 | 5 | 3 | 18115.7 | 5 | 5 | 3 | 17631.9 | 5 | 5 | 3 | 13011.4 | 5 | 5 | 3 | 11721.7 | 60480.6 |
| 5 | 5 | 4 | 18123.2 | 5 | 5 | 4 | 17627.8 | 5 | 5 | 4 | 13014.7 | 5 | 5 | 4 | 11716.2 | 60481.8 |
| 5 | 5 | 5 | 18120.7 | 5 | 5 | 5 | 17627.8 | 5 | 5 | 5 | 13012.8 | 5 | 5 | 5 | 11720.7 | 60482.0 |
| 5 | 5 | 6 | 18119.1 | 5 | 5 | 6 | 17633.5 | 5 | 5 | 6 | 13012.8 | 5 | 5 | 6 | 11726.3 | 60491.7 |
| 5 | 5 | 7 | 18118.9 | 5 | 5 | 7 | 17623.3 | 5 | 5 | 7 | 13027.0 | 5 | 5 | 7 | 11737.9 | 60507.1 |
| 5 | 5 | 8 | 18136.9 | 5 | 5 | 8 | 17630.4 | 5 | 5 | 8 | 13023.3 | 5 | 5 | 8 | 11754.3 | 60544.9 |
| 5 | 6 | 3 | 18148.9 | 5 | 6 | 3 | 17645.1 | 5 | 6 | 3 | 13010.8 | 5 | 6 | 3 | 11726.3 | 60531.0 |
| 5 | 6 | 4 | 18156.2 | 5 | 6 | 4 | 17641.0 | 5 | 6 | 4 | 13014.1 | 5 | 6 | 4 | 11720.8 | 60532.2 |
| 5 | 6 | 5 | 18153.7 | 5 | 6 | 5 | 17641.2 | 5 | 6 | 5 | 13012.3 | 5 | 6 | 5 | 11725.5 | 60532.6 |
| 5 | 6 | 6 | 18152.0 | 5 | 6 | 6 | 17646.6 | 5 | 6 | 6 | 13012.2 | 5 | 6 | 6 | 11730.9 | 60541.8 |
| 5 | 6 | 7 | 18152.1 | 5 | 6 | 7 | 17636.4 | 5 | 6 | 7 | 13026.6 | 5 | 6 | 7 | 11742.6 | 60557.8 |
| 5 | 6 | 8 | 18170.2 | 5 | 6 | 8 | 17643.6 | 5 | 6 | 8 | 13022.9 | 5 | 6 | 8 | 11759.6 | 60596.2 |
| 5 | 7 | 3 | 18155.0 | 5 | 7 | 3 | 17638.8 | 5 | 7 | 3 | 13030.4 | 5 | 7 | 3 | 11731.7 | 60555.9 |
| 5 | 7 | 4 | 18162.6 | 5 | 7 | 4 | 17634.8 | 5 | 7 | 4 | 13033.4 | 5 | 7 | 4 | 11726.1 | 60556.9 |
| 5 | 7 | 5 | 18160.1 | 5 | 7 | 5 | 17634.7 | 5 | 7 | 5 | 13031.5 | 5 | 7 | 5 | 11730.8 | 60557.1 |
| 5 | 7 | 6 | 18158.4 | 5 | 7 | 6 | 17640.3 | 5 | 7 | 6 | 13031.2 | 5 | 7 | 6 | 11736.2 | 60566.1 |
| 5 | 7 | 7 | 18158.5 | 5 | 7 | 7 | 17630.1 | 5 | 7 | 7 | 13045.7 | 5 | 7 | 7 | 11747.8 | 60582.1 |
| 5 | 7 | 8 | 18176.6 | 5 | 7 | 8 | 17637.1 | 5 | 7 | 8 | 13041.7 | 5 | 7 | 8 | 11765.0 | 60620.4 |
| 5 | 8 | 3 | 18151.7 | 5 | 8 | 3 | 17656.2 | 5 | 8 | 3 | 13034.9 | 5 | 8 | 3 | 11737.9 | 60580.7 |
| 5 | 8 | 4 | 18159.5 | 5 | 8 | 4 | 17652.1 | 5 | 8 | 4 | 13037.9 | 5 | 8 | 4 | 11732.5 | 60582.1 |
| 5 | 8 | 5 | 18157.1 | 5 | 8 | 5 | 17652.6 | 5 | 8 | 5 | 13035.9 | 5 | 8 | 5 | 11736.9 | 60582.5 |
| 5 | 8 | 6 | 18155.4 | 5 | 8 | 6 | 17657.9 | 5 | 8 | 6 | 13035.3 | 5 | 8 | 6 | 11742.4 | 60591.1 |
| 5 | 8 | 7 | 18155.4 | 5 | 8 | 7 | 17647.7 | 5 | 8 | 7 | 13049.4 | 5 | 8 | 7 | 11753.7 | 60606.2 |
| 5 | 8 | 8 | 18173.7 | 5 | 8 | 8 | 17654.9 | 5 | 8 | 8 | 13045.2 | 5 | 8 | 8 | 11770.9 | 60644.6 |
| 6 | 3 | 3 | 18107.7 | 6 | 3 | 3 | 17612.7 | 6 | 3 | 3 | 13003.9 | 6 | 3 | 3 | 11722.7 | 60447.0 |
| 6 | 3 | 4 | 18117.0 | 6 | 3 | 4 | 17608.8 | 6 | 3 | 4 | 13007.9 | 6 | 3 | 4 | 11717.2 | 60450.8 |
| 6 | 3 | 5 | 18114.7 | 6 | 3 | 5 | 17608.7 | 6 | 3 | 5 | 13006.2 | 6 | 3 | 5 | 11721.6 | 60451.2 |

| | | | | | | | | | | | | | | | | |
|---|---|---|---|---|---|---|---|---|---|---|---|---|---|---|---|---|
| 6 | 3 | 6 | 18112.5 | 6 | 3 | 6 | 17614.5 | 6 | 3 | 6 | 13006.5 | 6 | 3 | 6 | 11727.8 | 60461.3 |
| 6 | 3 | 7 | 18112.9 | 6 | 3 | 7 | 17604.3 | 6 | 3 | 7 | 13021.3 | 6 | 3 | 7 | 11740.0 | 60478.5 |
| 6 | 3 | 8 | 18131.2 | 6 | 3 | 8 | 17611.1 | 6 | 3 | 8 | 13017.8 | 6 | 3 | 8 | 11757.3 | 60517.4 |
| 6 | 4 | 3 | 18127.2 | 6 | 4 | 3 | 17620.4 | 6 | 4 | 3 | 13020.5 | 6 | 4 | 3 | 11721.0 | 60489.1 |
| 6 | 4 | 4 | 18135.4 | 6 | 4 | 4 | 17616.3 | 6 | 4 | 4 | 13023.5 | 6 | 4 | 4 | 11715.7 | 60490.9 |
| 6 | 4 | 5 | 18133.0 | 6 | 4 | 5 | 17616.2 | 6 | 4 | 5 | 13021.8 | 6 | 4 | 5 | 11719.9 | 60491.0 |
| 6 | 4 | 6 | 18131.1 | 6 | 4 | 6 | 17622.2 | 6 | 4 | 6 | 13022.0 | 6 | 4 | 6 | 11726.1 | 60501.3 |
| 6 | 4 | 7 | 18131.1 | 6 | 4 | 7 | 17611.9 | 6 | 4 | 7 | 13036.4 | 6 | 4 | 7 | 11738.3 | 60517.7 |
| 6 | 4 | 8 | 18149.5 | 6 | 4 | 8 | 17618.8 | 6 | 4 | 8 | 13032.6 | 6 | 4 | 8 | 11755.3 | 60556.2 |
| 6 | 5 | 3 | 18143.2 | 6 | 5 | 3 | 17629.5 | 6 | 5 | 3 | 13022.5 | 6 | 5 | 3 | 11723.6 | 60518.9 |
| 6 | 5 | 4 | 18150.6 | 6 | 5 | 4 | 17625.4 | 6 | 5 | 4 | 13025.4 | 6 | 5 | 4 | 11718.2 | 60519.6 |
| 6 | 5 | 5 | 18148.1 | 6 | 5 | 5 | 17625.6 | 6 | 5 | 5 | 13023.7 | 6 | 5 | 5 | 11723.0 | 60520.3 |
| 6 | 5 | 6 | 18146.4 | 6 | 5 | 6 | 17631.3 | 6 | 5 | 6 | 13023.8 | 6 | 5 | 6 | 11728.7 | 60530.2 |
| 6 | 5 | 7 | 18146.3 | 6 | 5 | 7 | 17621.0 | 6 | 5 | 7 | 13038.4 | 6 | 5 | 7 | 11741.1 | 60546.9 |
| 6 | 5 | 8 | 18164.5 | 6 | 5 | 8 | 17628.1 | 6 | 5 | 8 | 13034.6 | 6 | 5 | 8 | 11758.1 | 60585.3 |
| 6 | 6 | 3 | 18176.7 | 6 | 6 | 3 | 17642.7 | 6 | 6 | 3 | 13022.1 | 6 | 6 | 3 | 11728.2 | 60569.7 |
| 6 | 6 | 4 | 18184.0 | 6 | 6 | 4 | 17638.6 | 6 | 6 | 4 | 13025.1 | 6 | 6 | 4 | 11722.7 | 60570.3 |
| 6 | 6 | 5 | 18181.4 | 6 | 6 | 5 | 17638.9 | 6 | 6 | 5 | 13023.3 | 6 | 6 | 5 | 11727.7 | 60571.4 |
| 6 | 6 | 6 | 18179.7 | 6 | 6 | 6 | 17644.3 | 6 | 6 | 6 | 13023.4 | 6 | 6 | 6 | 11733.3 | 60580.7 |
| 6 | 6 | 7 | 18179.9 | 6 | 6 | 7 | 17634.1 | 6 | 6 | 7 | 13038.3 | 6 | 6 | 7 | 11745.8 | 60598.0 |
| 6 | 6 | 8 | 18198.1 | 6 | 6 | 8 | 17641.2 | 6 | 6 | 8 | 13034.4 | 6 | 6 | 8 | 11763.3 | 60637.0 |
| 6 | 7 | 3 | 18182.9 | 6 | 7 | 3 | 17636.2 | 6 | 7 | 3 | 13042.0 | 6 | 7 | 3 | 11733.7 | 60594.8 |
| 6 | 7 | 4 | 18190.4 | 6 | 7 | 4 | 17632.1 | 6 | 7 | 4 | 13044.7 | 6 | 7 | 4 | 11728.1 | 60595.4 |
| 6 | 7 | 5 | 18187.9 | 6 | 7 | 5 | 17632.3 | 6 | 7 | 5 | 13042.8 | 6 | 7 | 5 | 11733.2 | 60596.2 |
| 6 | 7 | 6 | 18186.1 | 6 | 7 | 6 | 17637.8 | 6 | 7 | 6 | 13042.7 | 6 | 7 | 6 | 11738.7 | 60605.3 |
| 6 | 7 | 7 | 18186.3 | 6 | 7 | 7 | 17627.6 | 6 | 7 | 7 | 13057.7 | 6 | 7 | 7 | 11751.1 | 60622.6 |
| 6 | 7 | 8 | 18204.6 | 6 | 7 | 8 | 17634.6 | 6 | 7 | 8 | 13053.5 | 6 | 7 | 8 | 11768.9 | 60661.5 |
| 6 | 8 | 3 | 18179.5 | 6 | 8 | 3 | 17653.7 | 6 | 8 | 3 | 13046.2 | 6 | 8 | 3 | 11740.1 | 60619.5 |
| 6 | 8 | 4 | 18187.2 | 6 | 8 | 4 | 17649.6 | 6 | 8 | 4 | 13048.9 | 6 | 8 | 4 | 11734.7 | 60620.4 |

| | | | | | | | | | | | | | | | | |
|---|---|---|---|---|---|---|---|---|---|---|---|---|---|---|---|---|
| 6 | 8 | 5 | 18184.8 | 6 | 8 | 5 | 17650.2 | 6 | 8 | 5 | 13047.0 | 6 | 8 | 5 | 11739.5 | 60621.4 |
| 6 | 8 | 6 | 18183.0 | 6 | 8 | 6 | 17655.5 | 6 | 8 | 6 | 13046.5 | 6 | 8 | 6 | 11745.1 | 60630.1 |
| 6 | 8 | 7 | 18183.1 | 6 | 8 | 7 | 17645.3 | 6 | 8 | 7 | 13061.0 | 6 | 8 | 7 | 11757.1 | 60646.5 |
| 6 | 8 | 8 | 18201.5 | 6 | 8 | 8 | 17652.4 | 6 | 8 | 8 | 13056.6 | 6 | 8 | 8 | 11775.0 | 60685.6 |
| 7 | 3 | 3 | 18088.2 | 7 | 3 | 3 | 17615.3 | 7 | 3 | 3 | 13013.5 | 7 | 3 | 3 | 11749.2 | 60466.2 |
| 7 | 3 | 4 | 18097.4 | 7 | 3 | 4 | 17611.2 | 7 | 3 | 4 | 13017.4 | 7 | 3 | 4 | 11743.7 | 60469.7 |
| 7 | 3 | 5 | 18095.0 | 7 | 3 | 5 | 17611.1 | 7 | 3 | 5 | 13015.8 | 7 | 3 | 5 | 11747.7 | 60469.7 |
| 7 | 3 | 6 | 18092.7 | 7 | 3 | 6 | 17616.7 | 7 | 3 | 6 | 13016.4 | 7 | 3 | 6 | 11754.5 | 60480.3 |
| 7 | 3 | 7 | 18092.9 | 7 | 3 | 7 | 17606.6 | 7 | 3 | 7 | 13032.0 | 7 | 3 | 7 | 11767.2 | 60498.6 |
| 7 | 3 | 8 | 18111.3 | 7 | 3 | 8 | 17613.4 | 7 | 3 | 8 | 13028.7 | 7 | 3 | 8 | 11784.5 | 60537.8 |
| 7 | 4 | 3 | 18107.6 | 7 | 4 | 3 | 17622.7 | 7 | 4 | 3 | 13030.1 | 7 | 4 | 3 | 11747.5 | 60508.0 |
| 7 | 4 | 4 | 18115.6 | 7 | 4 | 4 | 17618.6 | 7 | 4 | 4 | 13033.1 | 7 | 4 | 4 | 11742.1 | 60509.4 |
| 7 | 4 | 5 | 18113.2 | 7 | 4 | 5 | 17618.4 | 7 | 4 | 5 | 13031.4 | 7 | 4 | 5 | 11746.1 | 60509.1 |
| 7 | 4 | 6 | 18111.2 | 7 | 4 | 6 | 17624.2 | 7 | 4 | 6 | 13031.9 | 7 | 4 | 6 | 11752.7 | 60519.9 |
| 7 | 4 | 7 | 18110.9 | 7 | 4 | 7 | 17614.0 | 7 | 4 | 7 | 13047.1 | 7 | 4 | 7 | 11765.5 | 60537.5 |
| 7 | 4 | 8 | 18129.4 | 7 | 4 | 8 | 17620.9 | 7 | 4 | 8 | 13043.5 | 7 | 4 | 8 | 11782.7 | 60576.4 |
| 7 | 5 | 3 | 18123.5 | 7 | 5 | 3 | 17631.7 | 7 | 5 | 3 | 13031.9 | 7 | 5 | 3 | 11750.4 | 60537.5 |
| 7 | 5 | 4 | 18130.7 | 7 | 5 | 4 | 17627.5 | 7 | 5 | 4 | 13034.8 | 7 | 5 | 4 | 11744.9 | 60537.9 |
| 7 | 5 | 5 | 18128.2 | 7 | 5 | 5 | 17627.6 | 7 | 5 | 5 | 13033.1 | 7 | 5 | 5 | 11749.4 | 60538.2 |
| 7 | 5 | 6 | 18126.3 | 7 | 5 | 6 | 17633.1 | 7 | 5 | 6 | 13033.5 | 7 | 5 | 6 | 11755.6 | 60548.6 |
| 7 | 5 | 7 | 18126.0 | 7 | 5 | 7 | 17622.9 | 7 | 5 | 7 | 13048.9 | 7 | 5 | 7 | 11768.6 | 60566.4 |
| 7 | 5 | 8 | 18144.3 | 7 | 5 | 8 | 17629.9 | 7 | 5 | 8 | 13045.2 | 7 | 5 | 8 | 11785.8 | 60605.3 |
| 7 | 6 | 3 | 18156.8 | 7 | 6 | 3 | 17644.8 | 7 | 6 | 3 | 13031.5 | 7 | 6 | 3 | 11754.9 | 60588.0 |
| 7 | 6 | 4 | 18163.9 | 7 | 6 | 4 | 17640.6 | 7 | 6 | 4 | 13034.5 | 7 | 6 | 4 | 11749.3 | 60588.3 |
| 7 | 6 | 5 | 18161.3 | 7 | 6 | 5 | 17640.8 | 7 | 6 | 5 | 13032.7 | 7 | 6 | 5 | 11754.0 | 60588.9 |
| 7 | 6 | 6 | 18159.4 | 7 | 6 | 6 | 17646.1 | 7 | 6 | 6 | 13033.1 | 7 | 6 | 6 | 11760.2 | 60598.7 |
| 7 | 6 | 7 | 18159.4 | 7 | 6 | 7 | 17635.9 | 7 | 6 | 7 | 13048.7 | 7 | 6 | 7 | 11773.2 | 60617.2 |
| 7 | 6 | 8 | 18177.7 | 7 | 6 | 8 | 17643.0 | 7 | 6 | 8 | 13045.0 | 7 | 6 | 8 | 11790.9 | 60656.7 |
| 7 | 7 | 3 | 18163.0 | 7 | 7 | 3 | 17638.3 | 7 | 7 | 3 | 13051.2 | 7 | 7 | 3 | 11760.8 | 60613.3 |

| | | | | | | | | | | | | | | | | |
|---|---|---|---|---|---|---|---|---|---|---|---|---|---|---|---|---|
| 7 | 7 | 4 | 18170.4 | 7 | 7 | 4 | 17634.1 | 7 | 7 | 4 | 13053.8 | 7 | 7 | 4 | 11755.2 | 60613.5 |
| 7 | 7 | 5 | 18167.8 | 7 | 7 | 5 | 17634.2 | 7 | 7 | 5 | 13051.9 | 7 | 7 | 5 | 11759.9 | 60613.9 |
| 7 | 7 | 6 | 18165.9 | 7 | 7 | 6 | 17639.5 | 7 | 7 | 6 | 13052.1 | 7 | 7 | 6 | 11766.0 | 60623.5 |
| 7 | 7 | 7 | 18165.8 | 7 | 7 | 7 | 17629.4 | 7 | 7 | 7 | 13067.8 | 7 | 7 | 7 | 11778.9 | 60641.9 |
| 7 | 7 | 8 | 18184.3 | 7 | 7 | 8 | 17636.4 | 7 | 7 | 8 | 13063.9 | 7 | 7 | 8 | 11796.8 | 60681.4 |
| 7 | 8 | 3 | 18159.7 | 7 | 8 | 3 | 17655.6 | 7 | 8 | 3 | 13055.8 | 7 | 8 | 3 | 11768.2 | 60639.2 |
| 7 | 8 | 4 | 18167.2 | 7 | 8 | 4 | 17651.4 | 7 | 8 | 4 | 13058.5 | 7 | 8 | 4 | 11762.7 | 60639.8 |
| 7 | 8 | 5 | 18164.8 | 7 | 8 | 5 | 17652.0 | 7 | 8 | 5 | 13056.5 | 7 | 8 | 5 | 11767.1 | 60640.3 |
| 7 | 8 | 6 | 18162.8 | 7 | 8 | 6 | 17657.1 | 7 | 8 | 6 | 13056.3 | 7 | 8 | 6 | 11773.3 | 60649.5 |
| 7 | 8 | 7 | 18162.7 | 7 | 8 | 7 | 17646.9 | 7 | 8 | 7 | 13071.5 | 7 | 8 | 7 | 11785.9 | 60667.0 |
| 7 | 8 | 8 | 18181.3 | 7 | 8 | 8 | 17654.0 | 7 | 8 | 8 | 13067.4 | 7 | 8 | 8 | 11803.9 | 60706.6 |
| 8 | 3 | 3 | 18136.0 | 8 | 3 | 3 | 17618.7 | 8 | 3 | 3 | 13014.4 | 8 | 3 | 3 | 11746.4 | 60515.5 |
| 8 | 3 | 4 | 18145.1 | 8 | 3 | 4 | 17614.5 | 8 | 3 | 4 | 13018.3 | 8 | 3 | 4 | 11741.1 | 60519.0 |
| 8 | 3 | 5 | 18142.7 | 8 | 3 | 5 | 17614.4 | 8 | 3 | 5 | 13016.7 | 8 | 3 | 5 | 11744.8 | 60518.6 |
| 8 | 3 | 6 | 18139.8 | 8 | 3 | 6 | 17619.6 | 8 | 3 | 6 | 13017.4 | 8 | 3 | 6 | 11752.2 | 60529.0 |
| 8 | 3 | 7 | 18139.2 | 8 | 3 | 7 | 17609.6 | 8 | 3 | 7 | 13033.4 | 8 | 3 | 7 | 11766.0 | 60548.1 |
| 8 | 3 | 8 | 18158.0 | 8 | 3 | 8 | 17616.4 | 8 | 3 | 8 | 13030.2 | 8 | 3 | 8 | 11783.7 | 60588.3 |
| 8 | 4 | 3 | 18155.6 | 8 | 4 | 3 | 17626.1 | 8 | 4 | 3 | 13030.9 | 8 | 4 | 3 | 11744.8 | 60557.3 |
| 8 | 4 | 4 | 18163.5 | 8 | 4 | 4 | 17621.8 | 8 | 4 | 4 | 13033.9 | 8 | 4 | 4 | 11739.6 | 60558.7 |
| 8 | 4 | 5 | 18161.0 | 8 | 4 | 5 | 17621.7 | 8 | 4 | 5 | 13032.2 | 8 | 4 | 5 | 11743.2 | 60558.1 |
| 8 | 4 | 6 | 18158.3 | 8 | 4 | 6 | 17627.0 | 8 | 4 | 6 | 13032.8 | 8 | 4 | 6 | 11750.5 | 60568.6 |
| 8 | 4 | 7 | 18157.4 | 8 | 4 | 7 | 17616.9 | 8 | 4 | 7 | 13048.4 | 8 | 4 | 7 | 11764.3 | 60587.0 |
| 8 | 4 | 8 | 18176.2 | 8 | 4 | 8 | 17623.9 | 8 | 4 | 8 | 13044.9 | 8 | 4 | 8 | 11781.9 | 60626.9 |
| 8 | 5 | 3 | 18171.4 | 8 | 5 | 3 | 17634.9 | 8 | 5 | 3 | 13032.7 | 8 | 5 | 3 | 11747.6 | 60586.6 |
| 8 | 5 | 4 | 18178.4 | 8 | 5 | 4 | 17630.6 | 8 | 5 | 4 | 13035.7 | 8 | 5 | 4 | 11742.2 | 60587.0 |
| 8 | 5 | 5 | 18175.9 | 8 | 5 | 5 | 17630.7 | 8 | 5 | 5 | 13033.9 | 8 | 5 | 5 | 11746.3 | 60586.9 |
| 8 | 5 | 6 | 18173.4 | 8 | 5 | 6 | 17635.9 | 8 | 5 | 6 | 13034.4 | 8 | 5 | 6 | 11753.3 | 60597.0 |
| 8 | 5 | 7 | 18172.4 | 8 | 5 | 7 | 17625.8 | 8 | 5 | 7 | 13050.2 | 8 | 5 | 7 | 11767.4 | 60615.7 |
| 8 | 5 | 8 | 18191.0 | 8 | 5 | 8 | 17632.8 | 8 | 5 | 8 | 13046.7 | 8 | 5 | 8 | 11784.9 | 60655.5 |

| | | | | | | | | | | | | | | | | |
|---|---|---|---|---|---|---|---|---|---|---|---|---|---|---|---|---|
| 8 | 6 | 3 | 18204.7 | 8 | 6 | 3 | 17648.0 | 8 | 6 | 3 | 13032.4 | 8 | 6 | 3 | 11751.9 | 60636.9 |
| 8 | 6 | 4 | 18211.6 | 8 | 6 | 4 | 17643.7 | 8 | 6 | 4 | 13035.4 | 8 | 6 | 4 | 11746.5 | 60637.2 |
| 8 | 6 | 5 | 18209.0 | 8 | 6 | 5 | 17644.0 | 8 | 6 | 5 | 13033.6 | 8 | 6 | 5 | 11750.8 | 60637.4 |
| 8 | 6 | 6 | 18206.4 | 8 | 6 | 6 | 17648.8 | 8 | 6 | 6 | 13034.1 | 8 | 6 | 6 | 11757.6 | 60646.9 |
| 8 | 6 | 7 | 18205.6 | 8 | 6 | 7 | 17638.8 | 8 | 6 | 7 | 13050.1 | 8 | 6 | 7 | 11771.7 | 60666.2 |
| 8 | 6 | 8 | 18224.3 | 8 | 6 | 8 | 17645.9 | 8 | 6 | 8 | 13046.6 | 8 | 6 | 8 | 11789.8 | 60706.6 |
| 8 | 7 | 3 | 18210.6 | 8 | 7 | 3 | 17641.5 | 8 | 7 | 3 | 13052.0 | 8 | 7 | 3 | 11757.8 | 60661.9 |
| 8 | 7 | 4 | 18217.8 | 8 | 7 | 4 | 17637.2 | 8 | 7 | 4 | 13054.7 | 8 | 7 | 4 | 11752.4 | 60662.1 |
| 8 | 7 | 5 | 18215.2 | 8 | 7 | 5 | 17637.3 | 8 | 7 | 5 | 13052.9 | 8 | 7 | 5 | 11756.7 | 60662.1 |
| 8 | 7 | 6 | 18212.6 | 8 | 7 | 6 | 17642.3 | 8 | 7 | 6 | 13053.1 | 8 | 7 | 6 | 11763.4 | 60671.5 |
| 8 | 7 | 7 | 18211.9 | 8 | 7 | 7 | 17632.2 | 8 | 7 | 7 | 13069.2 | 8 | 7 | 7 | 11777.4 | 60690.7 |
| 8 | 7 | 8 | 18230.7 | 8 | 7 | 8 | 17639.3 | 8 | 7 | 8 | 13065.4 | 8 | 7 | 8 | 11795.7 | 60731.1 |
| 8 | 8 | 3 | 18207.3 | 8 | 8 | 3 | 17658.6 | 8 | 8 | 3 | 13056.8 | 8 | 8 | 3 | 11765.2 | 60687.9 |
| 8 | 8 | 4 | 18214.8 | 8 | 8 | 4 | 17654.3 | 8 | 8 | 4 | 13059.5 | 8 | 8 | 4 | 11759.9 | 60688.5 |
| 8 | 8 | 5 | 18212.3 | 8 | 8 | 5 | 17654.9 | 8 | 8 | 5 | 13057.5 | 8 | 8 | 5 | 11764.0 | 60688.7 |
| 8 | 8 | 6 | 18209.7 | 8 | 8 | 6 | 17659.6 | 8 | 8 | 6 | 13057.4 | 8 | 8 | 6 | 11770.8 | 60697.6 |
| 8 | 8 | 7 | 18208.8 | 8 | 8 | 7 | 17649.5 | 8 | 8 | 7 | 13073.0 | 8 | 8 | 7 | 11784.5 | 60715.9 |
| 8 | 8 | 8 | 18227.7 | 8 | 8 | 8 | 17656.7 | 8 | 8 | 8 | 13069.1 | 8 | 8 | 8 | 11802.9 | 60756.4 |

**Table S2.** Total quasi Akaike information criterion (qAIC) summed across all sites in **winter** by different combinations of knots and degrees of freedom for the spline function of years and day of the year (DOY). The lowest qAIC is in bold letters and it was for the combination: knots= 3, ns (year, df= 3), ns (DOY, df=3).

| **Stockholm-Winter** | | | | **Skåne-Winter** | | | | **Jämtland-Winter** | | | | **Västerbotten-Winter** | | | | **Total qAIC summed across all sites** |
|---|---|---|---|---|---|---|---|---|---|---|---|---|---|---|---|---|
| knots | ns (year, df) | ns (DOY, df) | qAIC | knots | ns (year, df) | ns (DOY, df) | qAIC | knots | ns (year, df) | ns (DOY, df) | qAIC | knots | ns (year, df) | ns (DOY, df) | qAIC | |
| 3 | 3 | 3 | 18036.6 | 3 | 3 | 3 | 16228.5 | 3 | 3 | 3 | 12240.9 | 3 | 3 | 3 | 12295.9 | **58801.9** |
| 3 | 3 | 4 | 18048.1 | 3 | 3 | 4 | 16238.9 | 3 | 3 | 4 | 12245.5 | 3 | 3 | 4 | 12303.9 | 58836.5 |
| 3 | 3 | 5 | 18125.7 | 3 | 3 | 5 | 16321.1 | 3 | 3 | 5 | 12260.2 | 3 | 3 | 5 | 12305.5 | 59012.5 |

| | | | | | | | | | | | | | | | | |
|---|---|---|---|---|---|---|---|---|---|---|---|---|---|---|---|---|
| 3 | 3 | 6 | 18159.3 | 3 | 3 | 6 | 16333.8 | 3 | 3 | 6 | 12263.9 | 3 | 3 | 6 | 12305.6 | 59062.6 |
| 3 | 3 | 7 | 18192.4 | 3 | 3 | 7 | 16341.1 | 3 | 3 | 7 | 12266.1 | 3 | 3 | 7 | 12302.9 | 59102.5 |
| 3 | 3 | 8 | 18224.3 | 3 | 3 | 8 | 16340.3 | 3 | 3 | 8 | 12269.5 | 3 | 3 | 8 | 12306.9 | 59141.0 |
| 3 | 4 | 3 | 18035.3 | 3 | 4 | 3 | 16286.6 | 3 | 4 | 3 | 12239.3 | 3 | 4 | 3 | 12299.4 | 58860.5 |
| 3 | 4 | 4 | 18046.8 | 3 | 4 | 4 | 16296.7 | 3 | 4 | 4 | 12243.8 | 3 | 4 | 4 | 12306.9 | 58894.2 |
| 3 | 4 | 5 | 18124.5 | 3 | 4 | 5 | 16386.0 | 3 | 4 | 5 | 12258.3 | 3 | 4 | 5 | 12308.0 | 59076.8 |
| 3 | 4 | 6 | 18158.1 | 3 | 4 | 6 | 16398.9 | 3 | 4 | 6 | 12261.9 | 3 | 4 | 6 | 12308.2 | 59127.1 |
| 3 | 4 | 7 | 18191.1 | 3 | 4 | 7 | 16406.2 | 3 | 4 | 7 | 12264.2 | 3 | 4 | 7 | 12305.6 | 59167.2 |
| 3 | 4 | 8 | 18222.9 | 3 | 4 | 8 | 16405.6 | 3 | 4 | 8 | 12267.7 | 3 | 4 | 8 | 12309.4 | 59205.6 |
| 3 | 5 | 3 | 18032.9 | 3 | 5 | 3 | 16283.9 | 3 | 5 | 3 | 12237.7 | 3 | 5 | 3 | 12311.1 | 58865.6 |
| 3 | 5 | 4 | 18044.4 | 3 | 5 | 4 | 16294.0 | 3 | 5 | 4 | 12242.3 | 3 | 5 | 4 | 12318.4 | 58899.1 |
| 3 | 5 | 5 | 18120.3 | 3 | 5 | 5 | 16384.3 | 3 | 5 | 5 | 12256.5 | 3 | 5 | 5 | 12319.6 | 59080.8 |
| 3 | 5 | 6 | 18153.8 | 3 | 5 | 6 | 16397.2 | 3 | 5 | 6 | 12260.1 | 3 | 5 | 6 | 12319.6 | 59130.7 |
| 3 | 5 | 7 | 18186.9 | 3 | 5 | 7 | 16404.6 | 3 | 5 | 7 | 12262.3 | 3 | 5 | 7 | 12317.2 | 59170.9 |
| 3 | 5 | 8 | 18218.8 | 3 | 5 | 8 | 16403.9 | 3 | 5 | 8 | 12265.8 | 3 | 5 | 8 | 12320.7 | 59209.2 |
| 3 | 6 | 3 | 18039.2 | 3 | 6 | 3 | 16283.5 | 3 | 6 | 3 | 12235.3 | 3 | 6 | 3 | 12308.3 | 58866.4 |
| 3 | 6 | 4 | 18050.6 | 3 | 6 | 4 | 16293.6 | 3 | 6 | 4 | 12239.9 | 3 | 6 | 4 | 12315.4 | 58899.5 |
| 3 | 6 | 5 | 18123.3 | 3 | 6 | 5 | 16383.7 | 3 | 6 | 5 | 12254.2 | 3 | 6 | 5 | 12316.3 | 59077.6 |
| 3 | 6 | 6 | 18156.4 | 3 | 6 | 6 | 16396.5 | 3 | 6 | 6 | 12257.9 | 3 | 6 | 6 | 12316.3 | 59127.1 |
| 3 | 6 | 7 | 18189.5 | 3 | 6 | 7 | 16403.9 | 3 | 6 | 7 | 12260.1 | 3 | 6 | 7 | 12313.9 | 59167.3 |
| 3 | 6 | 8 | 18221.6 | 3 | 6 | 8 | 16403.3 | 3 | 6 | 8 | 12263.5 | 3 | 6 | 8 | 12317.4 | 59205.9 |
| 3 | 7 | 3 | 18031.3 | 3 | 7 | 3 | 16328.3 | 3 | 7 | 3 | 12233.4 | 3 | 7 | 3 | 12307.8 | 58900.8 |
| 3 | 7 | 4 | 18042.8 | 3 | 7 | 4 | 16338.5 | 3 | 7 | 4 | 12238.0 | 3 | 7 | 4 | 12314.9 | 58934.3 |
| 3 | 7 | 5 | 18116.2 | 3 | 7 | 5 | 16427.0 | 3 | 7 | 5 | 12252.3 | 3 | 7 | 5 | 12316.1 | 59111.6 |
| 3 | 7 | 6 | 18149.6 | 3 | 7 | 6 | 16440.0 | 3 | 7 | 6 | 12255.9 | 3 | 7 | 6 | 12316.1 | 59161.6 |
| 3 | 7 | 7 | 18182.6 | 3 | 7 | 7 | 16447.7 | 3 | 7 | 7 | 12258.2 | 3 | 7 | 7 | 12313.7 | 59202.1 |
| 3 | 7 | 8 | 18214.8 | 3 | 7 | 8 | 16447.8 | 3 | 7 | 8 | 12261.8 | 3 | 7 | 8 | 12317.3 | 59241.8 |
| 3 | 8 | 3 | 18054.5 | 3 | 8 | 3 | 16377.4 | 3 | 8 | 3 | 12240.9 | 3 | 8 | 3 | 12315.2 | 58988.0 |
| 3 | 8 | 4 | 18065.9 | 3 | 8 | 4 | 16387.8 | 3 | 8 | 4 | 12245.2 | 3 | 8 | 4 | 12323.7 | 59022.6 |

| | | | | | | | | | | | | | | | | |
|---|---|---|---|---|---|---|---|---|---|---|---|---|---|---|---|---|
| 3 | 8 | 5 | 18145.0 | 3 | 8 | 5 | 16480.1 | 3 | 8 | 5 | 12259.2 | 3 | 8 | 5 | 12325.6 | 59209.9 |
| 3 | 8 | 6 | 18178.6 | 3 | 8 | 6 | 16493.5 | 3 | 8 | 6 | 12262.7 | 3 | 8 | 6 | 12325.4 | 59260.2 |
| 3 | 8 | 7 | 18211.6 | 3 | 8 | 7 | 16501.2 | 3 | 8 | 7 | 12264.9 | 3 | 8 | 7 | 12322.7 | 59300.3 |
| 3 | 8 | 8 | 18242.1 | 3 | 8 | 8 | 16501.3 | 3 | 8 | 8 | 12268.5 | 3 | 8 | 8 | 12326.8 | 59338.8 |
| 4 | 3 | 3 | 18048.0 | 4 | 3 | 3 | 16244.0 | 4 | 3 | 3 | 12237.1 | 4 | 3 | 3 | 12291.6 | 58820.7 |
| 4 | 3 | 4 | 18061.3 | 4 | 3 | 4 | 16254.7 | 4 | 3 | 4 | 12241.6 | 4 | 3 | 4 | 12299.9 | 58857.5 |
| 4 | 3 | 5 | 18140.9 | 4 | 3 | 5 | 16335.4 | 4 | 3 | 5 | 12256.1 | 4 | 3 | 5 | 12301.5 | 59033.8 |
| 4 | 3 | 6 | 18174.7 | 4 | 3 | 6 | 16346.7 | 4 | 3 | 6 | 12259.7 | 4 | 3 | 6 | 12301.8 | 59082.9 |
| 4 | 3 | 7 | 18208.8 | 4 | 3 | 7 | 16353.6 | 4 | 3 | 7 | 12261.9 | 4 | 3 | 7 | 12299.4 | 59123.7 |
| 4 | 3 | 8 | 18236.4 | 4 | 3 | 8 | 16351.9 | 4 | 3 | 8 | 12265.4 | 4 | 3 | 8 | 12302.9 | 59156.7 |
| 4 | 4 | 3 | 18046.7 | 4 | 4 | 3 | 16301.4 | 4 | 4 | 3 | 12235.5 | 4 | 4 | 3 | 12295.1 | 58878.6 |
| 4 | 4 | 4 | 18060.0 | 4 | 4 | 4 | 16311.8 | 4 | 4 | 4 | 12239.9 | 4 | 4 | 4 | 12302.8 | 58914.5 |
| 4 | 4 | 5 | 18139.7 | 4 | 4 | 5 | 16399.5 | 4 | 4 | 5 | 12254.2 | 4 | 4 | 5 | 12304.0 | 59097.3 |
| 4 | 4 | 6 | 18173.5 | 4 | 4 | 6 | 16411.0 | 4 | 4 | 6 | 12257.8 | 4 | 4 | 6 | 12304.4 | 59146.6 |
| 4 | 4 | 7 | 18207.5 | 4 | 4 | 7 | 16418.0 | 4 | 4 | 7 | 12260.1 | 4 | 4 | 7 | 12302.1 | 59187.6 |
| 4 | 4 | 8 | 18235.0 | 4 | 4 | 8 | 16416.4 | 4 | 4 | 8 | 12263.6 | 4 | 4 | 8 | 12305.4 | 59220.5 |
| 4 | 5 | 3 | 18044.6 | 4 | 5 | 3 | 16298.7 | 4 | 5 | 3 | 12233.9 | 4 | 5 | 3 | 12306.1 | 58883.3 |
| 4 | 5 | 4 | 18057.9 | 4 | 5 | 4 | 16309.1 | 4 | 5 | 4 | 12238.3 | 4 | 5 | 4 | 12313.7 | 58919.0 |
| 4 | 5 | 5 | 18135.7 | 4 | 5 | 5 | 16397.7 | 4 | 5 | 5 | 12252.4 | 4 | 5 | 5 | 12314.9 | 59100.7 |
| 4 | 5 | 6 | 18169.4 | 4 | 5 | 6 | 16409.3 | 4 | 5 | 6 | 12255.9 | 4 | 5 | 6 | 12315.0 | 59149.6 |
| 4 | 5 | 7 | 18203.4 | 4 | 5 | 7 | 16416.3 | 4 | 5 | 7 | 12258.2 | 4 | 5 | 7 | 12312.9 | 59190.7 |
| 4 | 5 | 8 | 18231.1 | 4 | 5 | 8 | 16414.6 | 4 | 5 | 8 | 12261.8 | 4 | 5 | 8 | 12316.1 | 59223.6 |
| 4 | 6 | 3 | 18050.7 | 4 | 6 | 3 | 16298.5 | 4 | 6 | 3 | 12231.5 | 4 | 6 | 3 | 12303.3 | 58884.0 |
| 4 | 6 | 4 | 18063.8 | 4 | 6 | 4 | 16308.9 | 4 | 6 | 4 | 12236.0 | 4 | 6 | 4 | 12310.6 | 58919.3 |
| 4 | 6 | 5 | 18138.5 | 4 | 6 | 5 | 16397.3 | 4 | 6 | 5 | 12250.1 | 4 | 6 | 5 | 12311.5 | 59097.4 |
| 4 | 6 | 6 | 18171.8 | 4 | 6 | 6 | 16408.8 | 4 | 6 | 6 | 12253.7 | 4 | 6 | 6 | 12311.7 | 59146.0 |
| 4 | 6 | 7 | 18205.9 | 4 | 6 | 7 | 16415.8 | 4 | 6 | 7 | 12255.9 | 4 | 6 | 7 | 12309.6 | 59187.1 |
| 4 | 6 | 8 | 18233.7 | 4 | 6 | 8 | 16414.3 | 4 | 6 | 8 | 12259.5 | 4 | 6 | 8 | 12312.7 | 59220.2 |
| 4 | 7 | 3 | 18042.8 | 4 | 7 | 3 | 16344.4 | 4 | 7 | 3 | 12229.6 | 4 | 7 | 3 | 12302.7 | 58919.6 |

| | | | | | | | | | | | | | | | | |
|---|---|---|---|---|---|---|---|---|---|---|---|---|---|---|---|---|
| 4 | 7 | 4 | 18056.1 | 4 | 7 | 4 | 16354.9 | 4 | 7 | 4 | 12234.0 | 4 | 7 | 4 | 12310.1 | 58955.2 |
| 4 | 7 | 5 | 18131.5 | 4 | 7 | 5 | 16441.8 | 4 | 7 | 5 | 12248.2 | 4 | 7 | 5 | 12311.1 | 59132.6 |
| 4 | 7 | 6 | 18165.0 | 4 | 7 | 6 | 16453.4 | 4 | 7 | 6 | 12251.8 | 4 | 7 | 6 | 12311.4 | 59181.6 |
| 4 | 7 | 7 | 18199.1 | 4 | 7 | 7 | 16460.7 | 4 | 7 | 7 | 12254.1 | 4 | 7 | 7 | 12309.2 | 59223.1 |
| 4 | 7 | 8 | 18227.0 | 4 | 7 | 8 | 16459.9 | 4 | 7 | 8 | 12257.7 | 4 | 7 | 8 | 12312.5 | 59257.1 |
| 4 | 8 | 3 | 18065.6 | 4 | 8 | 3 | 16393.8 | 4 | 8 | 3 | 12237.2 | 4 | 8 | 3 | 12310.2 | 59006.8 |
| 4 | 8 | 4 | 18078.8 | 4 | 8 | 4 | 16404.5 | 4 | 8 | 4 | 12241.4 | 4 | 8 | 4 | 12319.0 | 59043.6 |
| 4 | 8 | 5 | 18159.8 | 4 | 8 | 5 | 16495.1 | 4 | 8 | 5 | 12255.2 | 4 | 8 | 5 | 12320.8 | 59230.9 |
| 4 | 8 | 6 | 18193.5 | 4 | 8 | 6 | 16507.1 | 4 | 8 | 6 | 12258.7 | 4 | 8 | 6 | 12320.9 | 59280.1 |
| 4 | 8 | 7 | 18227.5 | 4 | 8 | 7 | 16514.3 | 4 | 8 | 7 | 12260.9 | 4 | 8 | 7 | 12318.4 | 59321.1 |
| 4 | 8 | 8 | 18253.8 | 4 | 8 | 8 | 16513.6 | 4 | 8 | 8 | 12264.6 | 4 | 8 | 8 | 12322.2 | 59354.1 |
| 5 | 3 | 3 | 18055.5 | 5 | 3 | 3 | 16263.0 | 5 | 3 | 3 | 12248.7 | 5 | 3 | 3 | 12305.8 | 58873.0 |
| 5 | 3 | 4 | 18067.8 | 5 | 3 | 4 | 16274.0 | 5 | 3 | 4 | 12253.3 | 5 | 3 | 4 | 12314.0 | 58909.0 |
| 5 | 3 | 5 | 18148.4 | 5 | 3 | 5 | 16351.7 | 5 | 3 | 5 | 12267.0 | 5 | 3 | 5 | 12316.0 | 59083.1 |
| 5 | 3 | 6 | 18182.8 | 5 | 3 | 6 | 16362.1 | 5 | 3 | 6 | 12270.5 | 5 | 3 | 6 | 12316.6 | 59131.9 |
| 5 | 3 | 7 | 18217.3 | 5 | 3 | 7 | 16368.4 | 5 | 3 | 7 | 12272.9 | 5 | 3 | 7 | 12314.4 | 59173.0 |
| 5 | 3 | 8 | 18246.2 | 5 | 3 | 8 | 16366.4 | 5 | 3 | 8 | 12276.3 | 5 | 3 | 8 | 12317.4 | 59206.4 |
| 5 | 4 | 3 | 18054.4 | 5 | 4 | 3 | 16321.6 | 5 | 4 | 3 | 12247.0 | 5 | 4 | 3 | 12309.4 | 58932.4 |
| 5 | 4 | 4 | 18066.7 | 5 | 4 | 4 | 16332.4 | 5 | 4 | 4 | 12251.4 | 5 | 4 | 4 | 12317.0 | 58967.6 |
| 5 | 4 | 5 | 18147.5 | 5 | 4 | 5 | 16417.0 | 5 | 4 | 5 | 12265.0 | 5 | 4 | 5 | 12318.6 | 59148.1 |
| 5 | 4 | 6 | 18181.9 | 5 | 4 | 6 | 16427.5 | 5 | 4 | 6 | 12268.4 | 5 | 4 | 6 | 12319.3 | 59197.1 |
| 5 | 4 | 7 | 18216.3 | 5 | 4 | 7 | 16433.9 | 5 | 4 | 7 | 12270.9 | 5 | 4 | 7 | 12317.2 | 59238.2 |
| 5 | 4 | 8 | 18245.1 | 5 | 4 | 8 | 16432.1 | 5 | 4 | 8 | 12274.3 | 5 | 4 | 8 | 12320.1 | 59271.6 |
| 5 | 5 | 3 | 18052.0 | 5 | 5 | 3 | 16319.3 | 5 | 5 | 3 | 12245.4 | 5 | 5 | 3 | 12320.7 | 58937.4 |
| 5 | 5 | 4 | 18064.4 | 5 | 5 | 4 | 16330.0 | 5 | 5 | 4 | 12249.9 | 5 | 5 | 4 | 12328.2 | 58972.4 |
| 5 | 5 | 5 | 18143.4 | 5 | 5 | 5 | 16415.6 | 5 | 5 | 5 | 12263.2 | 5 | 5 | 5 | 12329.7 | 59151.9 |
| 5 | 5 | 6 | 18177.7 | 5 | 5 | 6 | 16426.2 | 5 | 5 | 6 | 12266.6 | 5 | 5 | 6 | 12330.2 | 59200.6 |
| 5 | 5 | 7 | 18212.1 | 5 | 5 | 7 | 16432.5 | 5 | 5 | 7 | 12269.0 | 5 | 5 | 7 | 12328.3 | 59241.8 |
| 5 | 5 | 8 | 18241.0 | 5 | 5 | 8 | 16430.6 | 5 | 5 | 8 | 12272.5 | 5 | 5 | 8 | 12331.0 | 59275.1 |

| | | | | | | | | | | | | | | | | |
|---|---|---|---|---|---|---|---|---|---|---|---|---|---|---|---|---|
| 5 | 6 | 3 | 18057.8 | 5 | 6 | 3 | 16318.8 | 5 | 6 | 3 | 12243.1 | 5 | 6 | 3 | 12317.9 | 58937.6 |
| 5 | 6 | 4 | 18070.0 | 5 | 6 | 4 | 16329.5 | 5 | 6 | 4 | 12247.6 | 5 | 6 | 4 | 12325.0 | 58972.2 |
| 5 | 6 | 5 | 18145.9 | 5 | 6 | 5 | 16414.9 | 5 | 6 | 5 | 12261.0 | 5 | 6 | 5 | 12326.3 | 59148.1 |
| 5 | 6 | 6 | 18179.7 | 5 | 6 | 6 | 16425.5 | 5 | 6 | 6 | 12264.4 | 5 | 6 | 6 | 12326.8 | 59196.4 |
| 5 | 6 | 7 | 18214.2 | 5 | 6 | 7 | 16431.8 | 5 | 6 | 7 | 12266.8 | 5 | 6 | 7 | 12324.9 | 59237.7 |
| 5 | 6 | 8 | 18243.3 | 5 | 6 | 8 | 16430.0 | 5 | 6 | 8 | 12270.3 | 5 | 6 | 8 | 12327.6 | 59271.2 |
| 5 | 7 | 3 | 18050.4 | 5 | 7 | 3 | 16365.0 | 5 | 7 | 3 | 12241.1 | 5 | 7 | 3 | 12317.3 | 58973.8 |
| 5 | 7 | 4 | 18062.8 | 5 | 7 | 4 | 16375.8 | 5 | 7 | 4 | 12245.5 | 5 | 7 | 4 | 12324.6 | 59008.7 |
| 5 | 7 | 5 | 18139.4 | 5 | 7 | 5 | 16459.6 | 5 | 7 | 5 | 12258.9 | 5 | 7 | 5 | 12326.1 | 59184.0 |
| 5 | 7 | 6 | 18173.5 | 5 | 7 | 6 | 16470.3 | 5 | 7 | 6 | 12262.3 | 5 | 7 | 6 | 12326.6 | 59232.8 |
| 5 | 7 | 7 | 18207.9 | 5 | 7 | 7 | 16477.0 | 5 | 7 | 7 | 12264.8 | 5 | 7 | 7 | 12324.7 | 59274.4 |
| 5 | 7 | 8 | 18237.1 | 5 | 7 | 8 | 16475.9 | 5 | 7 | 8 | 12268.3 | 5 | 7 | 8 | 12327.5 | 59308.8 |
| 5 | 8 | 3 | 18072.8 | 5 | 8 | 3 | 16414.1 | 5 | 8 | 3 | 12249.0 | 5 | 8 | 3 | 12324.2 | 59060.1 |
| 5 | 8 | 4 | 18085.1 | 5 | 8 | 4 | 16425.1 | 5 | 8 | 4 | 12253.3 | 5 | 8 | 4 | 12332.8 | 59096.3 |
| 5 | 8 | 5 | 18167.4 | 5 | 8 | 5 | 16512.6 | 5 | 8 | 5 | 12266.3 | 5 | 8 | 5 | 12335.2 | 59281.4 |
| 5 | 8 | 6 | 18201.7 | 5 | 8 | 6 | 16523.7 | 5 | 8 | 6 | 12269.6 | 5 | 8 | 6 | 12335.5 | 59330.4 |
| 5 | 8 | 7 | 18236.1 | 5 | 8 | 7 | 16530.2 | 5 | 8 | 7 | 12272.0 | 5 | 8 | 7 | 12333.3 | 59371.6 |
| 5 | 8 | 8 | 18263.6 | 5 | 8 | 8 | 16529.2 | 5 | 8 | 8 | 12275.6 | 5 | 8 | 8 | 12336.5 | 59405.0 |
| 6 | 3 | 3 | 18051.5 | 6 | 3 | 3 | 16272.8 | 6 | 3 | 3 | 12251.5 | 6 | 3 | 3 | 12301.4 | 58877.2 |
| 6 | 3 | 4 | 18064.1 | 6 | 3 | 4 | 16283.9 | 6 | 3 | 4 | 12256.2 | 6 | 3 | 4 | 12310.2 | 58914.4 |
| 6 | 3 | 5 | 18144.7 | 6 | 3 | 5 | 16360.6 | 6 | 3 | 5 | 12269.9 | 6 | 3 | 5 | 12312.0 | 59087.2 |
| 6 | 3 | 6 | 18178.3 | 6 | 3 | 6 | 16370.6 | 6 | 3 | 6 | 12273.4 | 6 | 3 | 6 | 12312.6 | 59134.9 |
| 6 | 3 | 7 | 18213.0 | 6 | 3 | 7 | 16376.4 | 6 | 3 | 7 | 12275.8 | 6 | 3 | 7 | 12310.2 | 59175.4 |
| 6 | 3 | 8 | 18241.8 | 6 | 3 | 8 | 16374.4 | 6 | 3 | 8 | 12279.2 | 6 | 3 | 8 | 12313.3 | 59208.7 |
| 6 | 4 | 3 | 18050.4 | 6 | 4 | 3 | 16331.8 | 6 | 4 | 3 | 12249.9 | 6 | 4 | 3 | 12304.9 | 58937.0 |
| 6 | 4 | 4 | 18063.1 | 6 | 4 | 4 | 16342.7 | 6 | 4 | 4 | 12254.5 | 6 | 4 | 4 | 12313.1 | 58973.4 |
| 6 | 4 | 5 | 18143.9 | 6 | 4 | 5 | 16426.2 | 6 | 4 | 5 | 12267.9 | 6 | 4 | 5 | 12314.5 | 59152.6 |
| 6 | 4 | 6 | 18177.5 | 6 | 4 | 6 | 16436.4 | 6 | 4 | 6 | 12271.4 | 6 | 4 | 6 | 12315.2 | 59200.5 |
| 6 | 4 | 7 | 18212.1 | 6 | 4 | 7 | 16442.3 | 6 | 4 | 7 | 12273.9 | 6 | 4 | 7 | 12312.9 | 59241.1 |

| | | | | | | | | | | | | | | | | |
|---|---|---|---|---|---|---|---|---|---|---|---|---|---|---|---|---|
| 6 | 4 | 8 | 18240.8 | 6 | 4 | 8 | 16440.4 | 6 | 4 | 8 | 12277.3 | 6 | 4 | 8 | 12315.8 | 59274.3 |
| 6 | 5 | 3 | 18048.1 | 6 | 5 | 3 | 16329.4 | 6 | 5 | 3 | 12248.3 | 6 | 5 | 3 | 12315.8 | 58941.6 |
| 6 | 5 | 4 | 18060.8 | 6 | 5 | 4 | 16340.2 | 6 | 5 | 4 | 12252.9 | 6 | 5 | 4 | 12323.8 | 58977.6 |
| 6 | 5 | 5 | 18139.8 | 6 | 5 | 5 | 16424.8 | 6 | 5 | 5 | 12266.1 | 6 | 5 | 5 | 12325.2 | 59155.8 |
| 6 | 5 | 6 | 18173.3 | 6 | 5 | 6 | 16435.0 | 6 | 5 | 6 | 12269.6 | 6 | 5 | 6 | 12325.5 | 59203.4 |
| 6 | 5 | 7 | 18207.9 | 6 | 5 | 7 | 16440.8 | 6 | 5 | 7 | 12272.0 | 6 | 5 | 7 | 12323.5 | 59244.1 |
| 6 | 5 | 8 | 18236.7 | 6 | 5 | 8 | 16438.8 | 6 | 5 | 8 | 12275.5 | 6 | 5 | 8 | 12326.2 | 59277.2 |
| 6 | 6 | 3 | 18053.9 | 6 | 6 | 3 | 16328.8 | 6 | 6 | 3 | 12246.0 | 6 | 6 | 3 | 12312.9 | 58941.6 |
| 6 | 6 | 4 | 18066.5 | 6 | 6 | 4 | 16339.6 | 6 | 6 | 4 | 12250.6 | 6 | 6 | 4 | 12320.7 | 58977.4 |
| 6 | 6 | 5 | 18142.3 | 6 | 6 | 5 | 16424.0 | 6 | 6 | 5 | 12263.9 | 6 | 6 | 5 | 12321.8 | 59152.0 |
| 6 | 6 | 6 | 18175.4 | 6 | 6 | 6 | 16434.1 | 6 | 6 | 6 | 12267.4 | 6 | 6 | 6 | 12322.2 | 59199.1 |
| 6 | 6 | 7 | 18210.1 | 6 | 6 | 7 | 16439.9 | 6 | 6 | 7 | 12269.8 | 6 | 6 | 7 | 12320.2 | 59240.0 |
| 6 | 6 | 8 | 18239.1 | 6 | 6 | 8 | 16438.0 | 6 | 6 | 8 | 12273.2 | 6 | 6 | 8 | 12322.9 | 59273.2 |
| 6 | 7 | 3 | 18046.7 | 6 | 7 | 3 | 16374.4 | 6 | 7 | 3 | 12243.9 | 6 | 7 | 3 | 12312.3 | 58977.3 |
| 6 | 7 | 4 | 18059.4 | 6 | 7 | 4 | 16385.4 | 6 | 7 | 4 | 12248.4 | 6 | 7 | 4 | 12320.2 | 59013.4 |
| 6 | 7 | 5 | 18135.9 | 6 | 7 | 5 | 16468.2 | 6 | 7 | 5 | 12261.7 | 6 | 7 | 5 | 12321.5 | 59187.4 |
| 6 | 7 | 6 | 18169.3 | 6 | 7 | 6 | 16478.6 | 6 | 7 | 6 | 12265.2 | 6 | 7 | 6 | 12322.0 | 59235.1 |
| 6 | 7 | 7 | 18203.9 | 6 | 7 | 7 | 16484.7 | 6 | 7 | 7 | 12267.7 | 6 | 7 | 7 | 12319.9 | 59276.2 |
| 6 | 7 | 8 | 18233.0 | 6 | 7 | 8 | 16483.5 | 6 | 7 | 8 | 12271.2 | 6 | 7 | 8 | 12322.7 | 59310.4 |
| 6 | 8 | 3 | 18069.4 | 6 | 8 | 3 | 16424.3 | 6 | 8 | 3 | 12251.7 | 6 | 8 | 3 | 12319.2 | 59064.6 |
| 6 | 8 | 4 | 18082.0 | 6 | 8 | 4 | 16435.5 | 6 | 8 | 4 | 12256.1 | 6 | 8 | 4 | 12328.4 | 59102.0 |
| 6 | 8 | 5 | 18164.3 | 6 | 8 | 5 | 16522.0 | 6 | 8 | 5 | 12269.0 | 6 | 8 | 5 | 12330.5 | 59285.7 |
| 6 | 8 | 6 | 18197.8 | 6 | 8 | 6 | 16532.6 | 6 | 8 | 6 | 12272.4 | 6 | 8 | 6 | 12330.8 | 59333.6 |
| 6 | 8 | 7 | 18232.4 | 6 | 8 | 7 | 16538.7 | 6 | 8 | 7 | 12274.8 | 6 | 8 | 7 | 12328.4 | 59374.3 |
| 6 | 8 | 8 | 18259.8 | 6 | 8 | 8 | 16537.6 | 6 | 8 | 8 | 12278.4 | 6 | 8 | 8 | 12331.7 | 59407.5 |
| 7 | 3 | 3 | 18042.5 | 7 | 3 | 3 | 16283.3 | 7 | 3 | 3 | 12246.7 | 7 | 3 | 3 | 12316.3 | 58888.7 |
| 7 | 3 | 4 | 18055.7 | 7 | 3 | 4 | 16294.0 | 7 | 3 | 4 | 12251.4 | 7 | 3 | 4 | 12324.0 | 58925.2 |
| 7 | 3 | 5 | 18135.9 | 7 | 3 | 5 | 16370.4 | 7 | 3 | 5 | 12265.4 | 7 | 3 | 5 | 12325.2 | 59096.9 |
| 7 | 3 | 6 | 18168.9 | 7 | 3 | 6 | 16380.6 | 7 | 3 | 6 | 12268.9 | 7 | 3 | 6 | 12324.8 | 59143.2 |

| | | | | | | | | | | | | | | | | |
|---|---|---|---|---|---|---|---|---|---|---|---|---|---|---|---|---|
| 7 | 3 | 7 | 18203.2 | 7 | 3 | 7 | 16386.5 | 7 | 3 | 7 | 12271.3 | 7 | 3 | 7 | 12322.3 | 59183.4 |
| 7 | 3 | 8 | 18230.9 | 7 | 3 | 8 | 16385.3 | 7 | 3 | 8 | 12274.7 | 7 | 3 | 8 | 12326.1 | 59217.0 |
| 7 | 4 | 3 | 18041.3 | 7 | 4 | 3 | 16342.0 | 7 | 4 | 3 | 12245.1 | 7 | 4 | 3 | 12319.6 | 58948.0 |
| 7 | 4 | 4 | 18054.6 | 7 | 4 | 4 | 16352.5 | 7 | 4 | 4 | 12249.7 | 7 | 4 | 4 | 12326.8 | 58983.7 |
| 7 | 4 | 5 | 18134.9 | 7 | 4 | 5 | 16435.6 | 7 | 4 | 5 | 12263.5 | 7 | 4 | 5 | 12327.6 | 59161.7 |
| 7 | 4 | 6 | 18168.0 | 7 | 4 | 6 | 16446.0 | 7 | 4 | 6 | 12267.0 | 7 | 4 | 6 | 12327.3 | 59208.3 |
| 7 | 4 | 7 | 18202.2 | 7 | 4 | 7 | 16452.0 | 7 | 4 | 7 | 12269.4 | 7 | 4 | 7 | 12324.9 | 59248.5 |
| 7 | 4 | 8 | 18229.7 | 7 | 4 | 8 | 16450.9 | 7 | 4 | 8 | 12272.8 | 7 | 4 | 8 | 12328.6 | 59282.1 |
| 7 | 5 | 3 | 18039.0 | 7 | 5 | 3 | 16339.6 | 7 | 5 | 3 | 12243.4 | 7 | 5 | 3 | 12330.5 | 58952.6 |
| 7 | 5 | 4 | 18052.3 | 7 | 5 | 4 | 16350.0 | 7 | 5 | 4 | 12248.2 | 7 | 5 | 4 | 12337.5 | 58988.0 |
| 7 | 5 | 5 | 18130.9 | 7 | 5 | 5 | 16434.1 | 7 | 5 | 5 | 12261.7 | 7 | 5 | 5 | 12338.3 | 59164.9 |
| 7 | 5 | 6 | 18163.8 | 7 | 5 | 6 | 16444.5 | 7 | 5 | 6 | 12265.2 | 7 | 5 | 6 | 12337.7 | 59211.1 |
| 7 | 5 | 7 | 18198.0 | 7 | 5 | 7 | 16450.4 | 7 | 5 | 7 | 12267.6 | 7 | 5 | 7 | 12335.5 | 59251.5 |
| 7 | 5 | 8 | 18225.7 | 7 | 5 | 8 | 16449.3 | 7 | 5 | 8 | 12271.0 | 7 | 5 | 8 | 12339.0 | 59285.0 |
| 7 | 6 | 3 | 18045.0 | 7 | 6 | 3 | 16339.0 | 7 | 6 | 3 | 12241.2 | 7 | 6 | 3 | 12327.6 | 58952.8 |
| 7 | 6 | 4 | 18058.1 | 7 | 6 | 4 | 16349.5 | 7 | 6 | 4 | 12245.9 | 7 | 6 | 4 | 12334.4 | 58987.9 |
| 7 | 6 | 5 | 18133.5 | 7 | 6 | 5 | 16433.4 | 7 | 6 | 5 | 12259.5 | 7 | 6 | 5 | 12334.9 | 59161.3 |
| 7 | 6 | 6 | 18166.0 | 7 | 6 | 6 | 16443.8 | 7 | 6 | 6 | 12263.0 | 7 | 6 | 6 | 12334.3 | 59207.2 |
| 7 | 6 | 7 | 18200.4 | 7 | 6 | 7 | 16449.7 | 7 | 6 | 7 | 12265.4 | 7 | 6 | 7 | 12332.2 | 59247.6 |
| 7 | 6 | 8 | 18228.2 | 7 | 6 | 8 | 16448.7 | 7 | 6 | 8 | 12268.8 | 7 | 6 | 8 | 12335.6 | 59281.2 |
| 7 | 7 | 3 | 18037.6 | 7 | 7 | 3 | 16385.4 | 7 | 7 | 3 | 12239.0 | 7 | 7 | 3 | 12327.0 | 58988.9 |
| 7 | 7 | 4 | 18050.9 | 7 | 7 | 4 | 16395.9 | 7 | 7 | 4 | 12243.7 | 7 | 7 | 4 | 12333.8 | 59024.3 |
| 7 | 7 | 5 | 18126.9 | 7 | 7 | 5 | 16478.3 | 7 | 7 | 5 | 12257.3 | 7 | 7 | 5 | 12334.6 | 59197.1 |
| 7 | 7 | 6 | 18159.7 | 7 | 7 | 6 | 16488.8 | 7 | 7 | 6 | 12260.8 | 7 | 7 | 6 | 12334.1 | 59243.4 |
| 7 | 7 | 7 | 18194.0 | 7 | 7 | 7 | 16495.1 | 7 | 7 | 7 | 12263.3 | 7 | 7 | 7 | 12331.8 | 59284.2 |
| 7 | 7 | 8 | 18221.9 | 7 | 7 | 8 | 16494.8 | 7 | 7 | 8 | 12266.7 | 7 | 7 | 8 | 12335.4 | 59318.8 |
| 7 | 8 | 3 | 18060.3 | 7 | 8 | 3 | 16434.8 | 7 | 8 | 3 | 12246.8 | 7 | 8 | 3 | 12333.6 | 59075.5 |
| 7 | 8 | 4 | 18073.5 | 7 | 8 | 4 | 16445.5 | 7 | 8 | 4 | 12251.3 | 7 | 8 | 4 | 12341.7 | 59112.0 |
| 7 | 8 | 5 | 18155.2 | 7 | 8 | 5 | 16531.5 | 7 | 8 | 5 | 12264.5 | 7 | 8 | 5 | 12343.2 | 59294.4 |

| | | | | | | | | | | | | | | | | |
|---|---|---|---|---|---|---|---|---|---|---|---|---|---|---|---|---|
| 7 | 8 | 6 | 18188.1 | 7 | 8 | 6 | 16542.4 | 7 | 8 | 6 | 12267.9 | 7 | 8 | 6 | 12342.5 | 59341.0 |
| 7 | 8 | 7 | 18222.4 | 7 | 8 | 7 | 16548.6 | 7 | 8 | 7 | 12270.3 | 7 | 8 | 7 | 12340.0 | 59381.3 |
| 7 | 8 | 8 | 18248.7 | 7 | 8 | 8 | 16548.3 | 7 | 8 | 8 | 12273.9 | 7 | 8 | 8 | 12344.0 | 59414.9 |
| 8 | 3 | 3 | 18057.7 | 8 | 3 | 3 | 16304.8 | 8 | 3 | 3 | 12245.3 | 8 | 3 | 3 | 12324.6 | 58932.4 |
| 8 | 3 | 4 | 18071.0 | 8 | 3 | 4 | 16315.4 | 8 | 3 | 4 | 12250.0 | 8 | 3 | 4 | 12332.2 | 58968.6 |
| 8 | 3 | 5 | 18152.1 | 8 | 3 | 5 | 16391.7 | 8 | 3 | 5 | 12264.0 | 8 | 3 | 5 | 12334.0 | 59141.8 |
| 8 | 3 | 6 | 18185.2 | 8 | 3 | 6 | 16401.9 | 8 | 3 | 6 | 12267.7 | 8 | 3 | 6 | 12333.8 | 59188.7 |
| 8 | 3 | 7 | 18219.4 | 8 | 3 | 7 | 16407.9 | 8 | 3 | 7 | 12269.9 | 8 | 3 | 7 | 12331.3 | 59228.4 |
| 8 | 3 | 8 | 18246.7 | 8 | 3 | 8 | 16407.0 | 8 | 3 | 8 | 12273.3 | 8 | 3 | 8 | 12334.8 | 59261.7 |
| 8 | 4 | 3 | 18056.5 | 8 | 4 | 3 | 16363.2 | 8 | 4 | 3 | 12243.7 | 8 | 4 | 3 | 12327.9 | 58991.2 |
| 8 | 4 | 4 | 18069.9 | 8 | 4 | 4 | 16373.6 | 8 | 4 | 4 | 12248.3 | 8 | 4 | 4 | 12334.9 | 59026.6 |
| 8 | 4 | 5 | 18151.1 | 8 | 4 | 5 | 16456.6 | 8 | 4 | 5 | 12262.1 | 8 | 4 | 5 | 12336.3 | 59206.1 |
| 8 | 4 | 6 | 18184.3 | 8 | 4 | 6 | 16467.0 | 8 | 4 | 6 | 12265.7 | 8 | 4 | 6 | 12336.2 | 59253.3 |
| 8 | 4 | 7 | 18218.3 | 8 | 4 | 7 | 16473.1 | 8 | 4 | 7 | 12268.0 | 8 | 4 | 7 | 12333.7 | 59293.1 |
| 8 | 4 | 8 | 18245.5 | 8 | 4 | 8 | 16472.3 | 8 | 4 | 8 | 12271.4 | 8 | 4 | 8 | 12337.1 | 59326.2 |
| 8 | 5 | 3 | 18054.4 | 8 | 5 | 3 | 16360.8 | 8 | 5 | 3 | 12242.0 | 8 | 5 | 3 | 12339.4 | 58996.5 |
| 8 | 5 | 4 | 18067.7 | 8 | 5 | 4 | 16371.1 | 8 | 5 | 4 | 12246.7 | 8 | 5 | 4 | 12346.2 | 59031.7 |
| 8 | 5 | 5 | 18147.0 | 8 | 5 | 5 | 16455.1 | 8 | 5 | 5 | 12260.3 | 8 | 5 | 5 | 12347.6 | 59210.1 |
| 8 | 5 | 6 | 18180.1 | 8 | 5 | 6 | 16465.5 | 8 | 5 | 6 | 12263.9 | 8 | 5 | 6 | 12347.3 | 59256.8 |
| 8 | 5 | 7 | 18214.2 | 8 | 5 | 7 | 16471.5 | 8 | 5 | 7 | 12266.2 | 8 | 5 | 7 | 12344.9 | 59296.8 |
| 8 | 5 | 8 | 18241.5 | 8 | 5 | 8 | 16470.6 | 8 | 5 | 8 | 12269.6 | 8 | 5 | 8 | 12348.1 | 59329.9 |
| 8 | 6 | 3 | 18060.7 | 8 | 6 | 3 | 16360.4 | 8 | 6 | 3 | 12239.8 | 8 | 6 | 3 | 12336.5 | 58997.3 |
| 8 | 6 | 4 | 18073.9 | 8 | 6 | 4 | 16370.7 | 8 | 6 | 4 | 12244.5 | 8 | 6 | 4 | 12343.0 | 59032.1 |
| 8 | 6 | 5 | 18150.0 | 8 | 6 | 5 | 16454.5 | 8 | 6 | 5 | 12258.2 | 8 | 6 | 5 | 12344.1 | 59206.8 |
| 8 | 6 | 6 | 18182.6 | 8 | 6 | 6 | 16464.9 | 8 | 6 | 6 | 12261.8 | 8 | 6 | 6 | 12343.8 | 59253.2 |
| 8 | 6 | 7 | 18216.8 | 8 | 6 | 7 | 16470.9 | 8 | 6 | 7 | 12264.0 | 8 | 6 | 7 | 12341.5 | 59293.3 |
| 8 | 6 | 8 | 18244.2 | 8 | 6 | 8 | 16470.2 | 8 | 6 | 8 | 12267.4 | 8 | 6 | 8 | 12344.7 | 59326.4 |
| 8 | 7 | 3 | 18053.0 | 8 | 7 | 3 | 16406.8 | 8 | 7 | 3 | 12237.6 | 8 | 7 | 3 | 12335.9 | 59033.3 |
| 8 | 7 | 4 | 18066.4 | 8 | 7 | 4 | 16417.3 | 8 | 7 | 4 | 12242.2 | 8 | 7 | 4 | 12342.6 | 59068.5 |

| | | | | | | | | | | | | | | | | |
|---|---|---|---|---|---|---|---|---|---|---|---|---|---|---|---|---|
| 8 | 7 | 5 | 18143.1 | 8 | 7 | 5 | 16499.5 | 8 | 7 | 5 | 12255.9 | 8 | 7 | 5 | 12344.0 | 59242.5 |
| 8 | 7 | 6 | 18176.0 | 8 | 7 | 6 | 16510.1 | 8 | 7 | 6 | 12259.5 | 8 | 7 | 6 | 12343.7 | 59289.4 |
| 8 | 7 | 7 | 18210.1 | 8 | 7 | 7 | 16516.5 | 8 | 7 | 7 | 12261.9 | 8 | 7 | 7 | 12341.3 | 59329.8 |
| 8 | 7 | 8 | 18237.7 | 8 | 7 | 8 | 16516.5 | 8 | 7 | 8 | 12265.3 | 8 | 7 | 8 | 12344.6 | 59364.0 |
| 8 | 8 | 3 | 18075.8 | 8 | 8 | 3 | 16456.1 | 8 | 8 | 3 | 12245.5 | 8 | 8 | 3 | 12342.6 | 59120.0 |
| 8 | 8 | 4 | 18089.1 | 8 | 8 | 4 | 16466.7 | 8 | 8 | 4 | 12249.9 | 8 | 8 | 4 | 12350.5 | 59156.3 |
| 8 | 8 | 5 | 18171.6 | 8 | 8 | 5 | 16552.6 | 8 | 8 | 5 | 12263.2 | 8 | 8 | 5 | 12352.7 | 59340.1 |
| 8 | 8 | 6 | 18204.6 | 8 | 8 | 6 | 16563.5 | 8 | 8 | 6 | 12266.7 | 8 | 8 | 6 | 12352.3 | 59387.2 |
| 8 | 8 | 7 | 18238.7 | 8 | 8 | 7 | 16569.8 | 8 | 8 | 7 | 12269.0 | 8 | 8 | 7 | 12349.6 | 59427.1 |
| 8 | 8 | 8 | 18264.6 | 8 | 8 | 8 | 16569.8 | 8 | 8 | 8 | 12272.5 | 8 | 8 | 8 | 12353.3 | 59460.3 |
